# Heat Exposure Definitions in Temperature–Mortality Studies: Towards a Harmonisation Framework

**DOI:** 10.1007/s40572-026-00571-5

**Published:** 2026-10-23

**Authors:** Aurelio Tobías, Masahiro Hashizume, Lina Madaniyazi

**Affiliations:** 1 Institute of Environmental Assessment and Water Research (IDAEA), Spanish National Council for Scientific Research (CSIC), Barcelona, Spain; 2 School of Tropical Medicine and Global Health, Nagasaki University, Nagasaki, Japan; 3 Department of Global Health Policy, Graduate School of Medicine, The University of Tokyo, Tokyo, Japan

**Keywords:** Temperature, Heat, Thresholds, Mortality, Distributed lag non-linear model, Exposure–response, Heat health warning systems

## Abstract

**Purpose of the Review:**

We reviewed how heat exposure is defined using thresholds and reference temperatures derived from the temperature–mortality exposure–response curve in time-series and case-crossover studies using distributed lag non-linear models and propose a harmonisation framework relevant to climate change research and heat disaster preparedness.

**Recent Findings:**

The rapid review identified variation across studies in heat thresholds and reference temperatures, affecting the comparability of estimates. A case study in Spain illustrates how the minimum mortality temperature (MMT) approach estimates overall heat-related mortality and its contributions from moderate and extreme heat, whereas the percentile-based approach estimates excessive heat mortality that could potentially be prevented by protective measures operating above the heat threshold.

**Summary:**

Differences in heat thresholds and reference temperatures make findings harder to compare across studies and complicate risk communication. Explicit reporting of these two components and their relevance to the research question and intended application would improve interpretation and translation to public health action.

**Supplementary Information:**

The online version contains supplementary material available at https://doi.org/10.1007/s40572-026-00571-5.

## Introduction

A large body of scientific evidence has established the harmful impacts of increased temperatures on human health. Mortality is a key outcome for assessing the population burden of heat and informing public health prevention [1–3]. In the context of climate change, these risks are expected to intensify as heat extremes become more frequent, more intense, and more persistent [4]. According to the Copernicus Climate Change Service, global temperatures in 2024 were approximately 1.6 °C above pre-industrial levels, and the average for 2023–2025 exceeded 1.5 °C for the first time [5, 6]. Warming differs across regions, with Europe warming more than twice as fast as the global average [7]. An estimated 61,672 heat-related deaths occurred in Europe during the summer of 2022, highlighting the substantial mortality burden associated with high temperatures [8]. This has contributed to the recognition of extreme heat as an environmental disaster risk, requiring preparedness plans, early warning systems, and coordinated public health responses [9, 10].

Despite strong evidence that high temperatures adversely affect health, studies use different thresholds to define heat exposure and reference temperatures to estimate mortality risks and attributable burdens from the temperature–mortality exposure–response curve. These temperature thresholds should be distinguished from the exposure metric used to measure heat, such as daily mean, maximum, or apparent temperature [11, 12]. The choice of heat thresholds and reference temperatures determines the exposure contrasts and counterfactual scenarios used in these comparisons [13]. These choices may address different research questions, but their implications are not always made explicit. Using the minimum mortality temperature (MMT) as the reference temperature quantifies heat-related mortality relative to the lowest estimated mortality risk, whereas using an activation threshold as the reference temperature quantifies the additional burden associated with temperatures above the point at which specified protective measures are activated. These estimates answer complementary questions: the former characterises the overall heat-related burden, whereas the latter provides an estimate of the potentially preventable burden for interventions activated above an activation threshold.

When these distinctions are not clearly stated, heat effects become harder to compare across settings and findings harder to synthesise. These unclear distinctions also complicate risk communication with the media, the public, and policymakers, who may not be familiar with epidemiological modelling choices. Ultimately, this may weaken the design and evaluation of heat health warning systems and heat action plans, which are central to heat disaster preparedness.

We combine a rapid review with a case study in Spain to address three questions. First, what heat thresholds and reference temperatures are used to characterise heat exposure contrasts in temperature–mortality studies. Second, how do these choices affect the interpretation of mortality risks and attributable burdens when applied to the same data. Finally, how can these findings inform a harmonisation framework that makes the analytical choices explicit while accommodating different epidemiological objectives and public health applications. The proposed framework aims to improve comparability and interpretation without requiring identical thresholds across geographical or population contexts.

## Methods

### Conceptual Framework

**Fig. 1:**
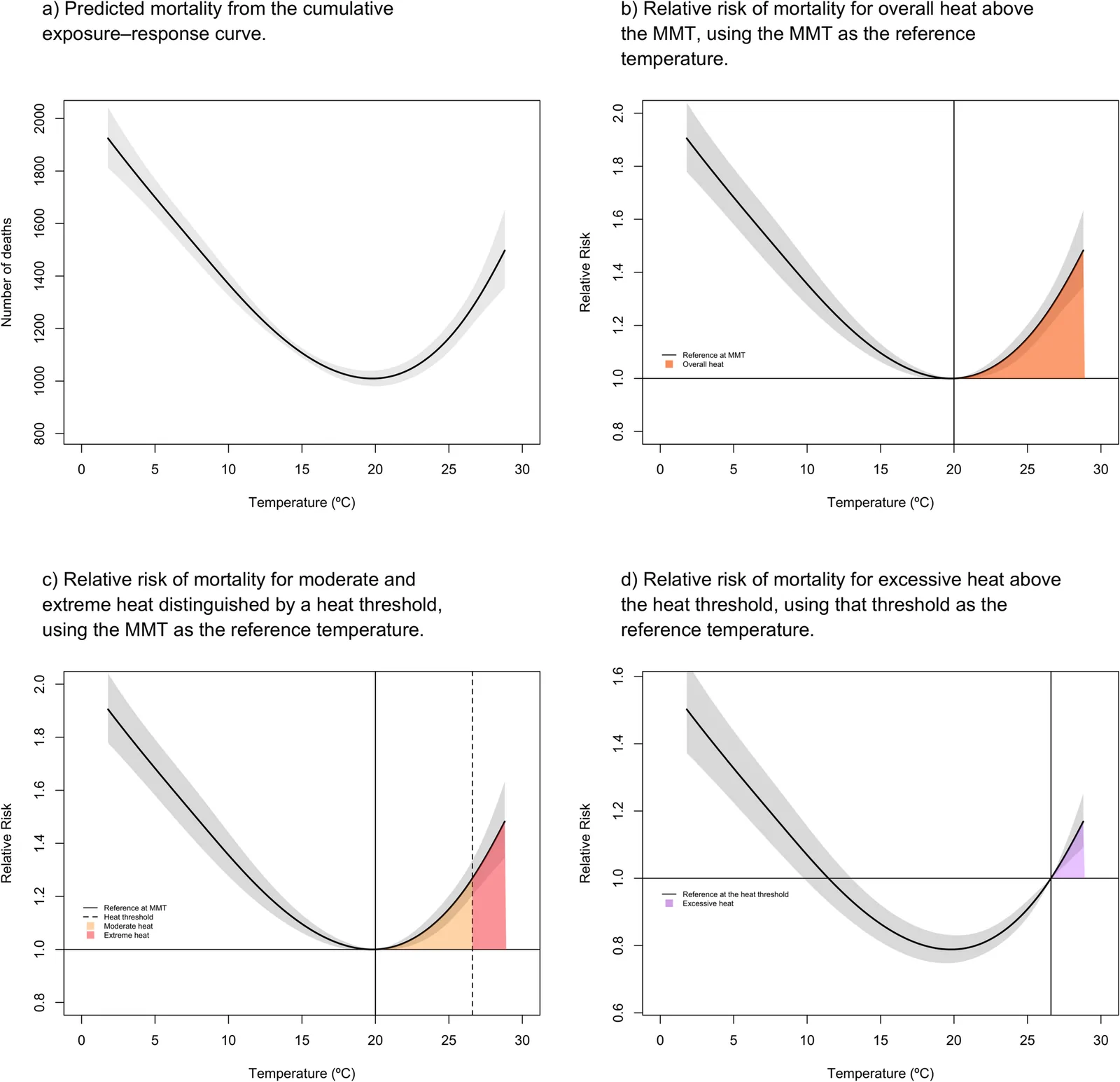
Conceptual framework for estimating heat-related mortality using different heat exposure thresholds and reference temperatures

The relationship between temperature and mortality is characterised by a non-linear association, often U- or V-shaped [14]. As illustrated in Fig. 1a, predicted mortality counts reveal that both excessively high and low temperatures are associated with increased mortality. Although splines provide flexibility for modelling this non-linear association, the mortality risk cannot be summarised by a single effect estimate across the temperature range. Its estimation therefore requires a reference temperature against which mortality at other temperatures is compared.

The MMT approach takes the temperature associated with the lowest estimated mortality as the reference temperature to quantify the overall heat-related mortality attributable to temperatures above the MMT (Fig. 1b) [15]. This burden can be divided into contributions from moderate and extreme heat by selecting an additional arbitrary extreme heat threshold (Fig. 1c) [1]. Moderate heat comprises temperatures between the MMT and this threshold, while extreme heat comprises temperatures above it. Both contributions are estimated relative to the MMT.

Alternatively, in the percentile-based approach, the temperature–mortality exposure–response curve is centred at a selected percentile of the temperature distribution (Fig. 1d). This reference temperature is often selected to correspond to the activation threshold at which a heat health warning system activates protective measures. The estimated heat-related deaths attributable to temperatures above the activation threshold are those that could potentially be prevented by these measures. We could refer to heat above this threshold as excessive heat, with heat-related mortality estimated relative to the risk at that threshold. Mortality attributable to temperatures below that threshold would remain outside the scope of the preventive measures.

Therefore, estimating heat-related mortality is characterised by two choices: the thresholds used to define overall heat exposure and, where applicable, distinguish moderate from extreme heat, and the reference temperature, which provides the baseline mortality risk. This framework guided the synthesis in the rapid review and the analysis in the case study.

### Part 1. Literature Review

#### Search Strategy

We conducted a rapid review to characterise how heat exposure thresholds and reference temperatures have been used in time-series and case-crossover studies using distributed lag non-linear models (DLNMs). Reporting was guided by the interim guidance for rapid reviews [16]. The completed PRISMA 2020 checklist is provided in the Supplementary Appendix. The review protocol was registered in Zenodo (https://doi.org/10.5281/zenodo.23307902).

We searched PubMed via the National Library of Medicine interface using terms covering heat, mortality, DLNMs, and the relevant study designs. We included only studies whose titles explicitly referred to mortality and to heat, extreme heat, high temperatures, or non-optimal temperatures. The search covered publications available up to 31 December 2025, and no language restrictions were applied to the search. The search strategy is provided in Supplementary Table 1. Restricting the search to PubMed and requiring temperature-related and mortality terms in titles provided a focused and manageable search for this methodological review, although these restrictions may have reduced its comprehensiveness.

Only original research articles were eligible for inclusion. Eligible studies examined associations between daily heat exposure and mortality in human populations using DLNMs. We included all-cause, non-accidental, and cause-specific mortality outcomes and considered different exposure metrics, including daily mean, maximum, and minimum temperature and composite thermal indices. Titles and abstracts were screened, followed by assessment of potentially eligible full texts. Studies that did not examine daily temperature or thermal indices, focused on other environmental stressors, or were not conducted in humans were excluded.

#### Study Selection and Data Extraction

Study screening and data extraction were conducted independently by two reviewers, with disagreements resolved through discussion with a third reviewer. From each included study, we extracted the setting, study period, study design, mortality outcome, temperature metric, lag window, and how heat was defined from the temperature–mortality exposure–response curve, including the reference temperature at which it was centred, the threshold used for heat and, where applicable, moderate and extreme heat, and the scale used to express thresholds. For studies reporting multiple mortality outcomes, temperature metrics, or heat exposure definitions, all were extracted. The information extracted from each study is presented in Supplementary Table 2.

#### Synthesis Approach

We examined the transparency and reproducibility of reporting by assessing whether studies clearly stated the reference temperature, justified the heat threshold in relation to the temperature–mortality exposure–response curve, and reported the lag structure. Neither risk of bias nor certainty of evidence was formally assessed, as this rapid review focused on characterising analytical definitions rather than synthesising evidence on temperature–mortality associations.

We conducted a narrative synthesis structured around recurring definition strategies. We grouped studies according to the heat exposure threshold and reference temperature, distinguishing approaches based on the MMT. We distinguished between percentile-based and absolute temperature thresholds and identified studies reporting more than one heat definition. Study characteristics were summarised descriptively, distinguishing study-level counts from counts of multiple outcomes, temperature metrics, heat thresholds and reference temperatures.

### Part 2. Case Study

#### Data

We analysed the temperature–mortality association in Spain between 2015 and 2022. Daily all-cause mortality data were obtained from the Spanish Daily Mortality Monitoring System (MoMo), available through open access since 2015. Daily mean temperature was calculated from observations at the reference stations of the Spanish State Meteorological Agency (AEMET) OpenData platform [17].

#### Statistical Analysis

We applied a standard time-series quasi-Poisson regression using a model specification evaluated through sensitivity analyses in previously published temperature–mortality studies in Spain using the same data [15, 18]. We fitted a natural cubic spline of time with 10 degrees of freedom (df) per year to control for seasonal and long-term trends, along with an indicator for the day of the week. We estimated the temperature-mortality association using a distributed lag non-linear model. Specifically, we modelled the temperature–mortality exposure–response curve with a natural cubic spline featuring three internal knots placed at the 10th, 75th, and 90th percentiles of the temperature distribution. The lag-response curve was modelled over 21 days using a natural cubic spline with three internal knots at equally spaced values on the log scale.

#### Mortality Risk and Attributable Mortality Estimation

We estimated the mortality risk and attributable burden for the summer of 2022, from June 1 to August 31, from the overall cumulative temperature–mortality exposure–response curve under two approaches. First, the MMT approach centred the curve at the MMT and distinguished between overall heat, moderate heat between the MMT and the 97.5th percentile of the annual temperature distribution, and extreme heat above the 97.5th percentile. We calculated the corresponding relative risk and attributable mortality for each heat definition. Second, the percentile-based approach centred the curve at the 97.5th percentile to estimate the relative risk and attributable mortality associated with temperatures above this percentile, providing an estimate of the potentially preventable burden for interventions activated at that point.

The choice of the annual 97.5th percentile was made because, in our data, it corresponded approximately to the 95th percentile of the summer temperature distribution, between June-September, used in the Spanish National Heat Health Prevention Plan [19].

## Results

### Part 1. Literature Review

#### Study Selection and Characteristics

The search identified 78 studies, of which we screened titles and abstracts, and we assessed 55 for eligibility. We included 41 studies in the final synthesis (Supplementary Fig. 1).

Studies were mainly conducted in Asia and Europe (39% each), with a small number in the Americas and Australia (Table 1). All-cause or non-accidental mortality accounted for 26.5% of the mortality outcomes extracted, whereas cardiovascular mortality accounted for 14.7% and respiratory mortality for 9.8%. More specific outcomes accounted for 49.0%, ranging from specific cardiovascular causes to heat-related illness. Daily mean temperature was the most used exposure metric (53.8%), although some studies also considered daily maximum temperature (16.9%), daily minimum temperature (12.3%), or other indices (16.9%).

**Table 1:** Characteristics of the studies included in the rapid review

Characteristic	*n*	(%)
**Region**		
Asia	16	(39.0)
Europe	16	(39.0)
America	4	(9.8)
Worldwide	3	(7.3)
Australia	2	(4.9)
**Study design**		
Time-series	20	(48.8)
Case-crossover	13	(31.7)
Time-series with GAM	5	(12.2)
Case time-series	3	(7.3)
Mortality outcome ^*^		
All-cause/non-accidental	27	(26.5)
All cardiovascular causes	15	(14.7)
All respiratory causes	10	(9.8)
Cause-specific	50	(49.0)
Temperature metrics ^*^		
Mean temperature	35	(53.8)
Maximum temperature	11	(16.9)
Minimum temperature	8	(12.3)
Other indices	11	(16.9)

^*^Some studies reported multiple mortality outcomes and temperature metrics.

#### Reference Temperatures

The MMT was the most frequently reported reference temperature, appearing in 27 of the 41 studies (65.9%) (Table 2). MMT values were either reported as a single temperature or estimated separately by geographical area, population subgroup, mortality cause or study period. Ten studies selected a percentile of the temperature distribution, including the 50th percentile in six studies (14.6%), the 75th percentile in three (7.3%) and the 90th percentile in one (2.4%). Three studies (7.3%) selected an absolute temperature value, while one study (2.4%) did not report the reference temperature.

**Table 2:** Reference temperatures used to estimate heat-related mortality in the studies included in the rapid review

Reference temperature	*n*	(%)
MMT	27	(65.9)
50th percentile	6	(14.6)
75th percentile	3	(7.3)
90th percentile	1	(2.4)
Temperature value (16 °C, 22 °C, 28 °C)	3	(7.3)
Not reported	1	(2.4)

#### Heat Exposure Definitions

The 35 studies reporting mortality risks contributed 54 heat exposure definitions, while the 19 studies reporting attributable mortality contributed 34 definitions. Studies could report both mortality effect measures and more than one heat exposure definition for each measure (Table 3).

**Table 3:** Heat exposure definitions used to estimate mortality risks and attributable mortality in the studies included in the rapid review

Mortality effect measure/ Heat exposure definition^*^	*n*	(%)
Mortality risk (*n* = 54)		
Heat	43	(79.6)
At the 99th percentile	18	(33.3)
At the 95th percentile	6	(11.1)
At the 90th percentile	5	(9.3)
At the maximum temperature	1	(1.9)
At other percentiles (84.1st, 92.5th, 97.5th, 97.7th, 98th, 99.5th)	7	(13.0)
At a temperature value (29 °C, 35 °C, 37 °C)	4	(7.4)
Others	2	(3.7)
Moderate heat	5	(9.3)
At the 90th percentile	3	(5.6)
At the 95th percentile	1	(1.9)
At the 97.5th percentile	1	(1.9)
Extreme heat	6	(11.1)
At the 97.5th percentile	1	(1.9)
At the 99th percentile	5	(9.3)
Attributable mortality (*n* = 34)		
Heat	21	(61.8)
> MMT	11	(32.4)
> A percentile (50th, 97.5th)	3	(8.8)
> A temperature value (20 °C, 34 °C, 37 °C)	3	(8.8)
Others	4	(11.8)
Moderate heat	6	(17.6)
Between the MMT and 97th or 97.5th percentile	4	(11.8)
Between the MMT and 99th percentile	1	(2.9)
Between the 50th and 97.5th percentiles	1	(2.9)
Extreme heat	7	(20.6)
> 95th percentile	1	(2.9)
> 97th or 97.5th percentile	5	(14.7)
> 99th percentile	1	(2.9)

^*^Some studies reported multiple mortality effect measures and heat exposure definitions.

For mortality risk, 43 definitions (79.6%) described heat without explicitly identifying it as moderate or extreme. Comparisons at the 99th percentile were the most frequent, accounting for 18 definitions (33.3% of all risk definitions), followed by the 95th percentile (11.1%) and the 90th percentile (9.3%). Other definitions involved different percentiles, absolute temperature values, the maximum temperature or alternative exposure summaries. Five definitions (9.3%) explicitly described moderate heat, most frequently at the 90th percentile, while six (11.1%) described extreme heat, predominantly at the 99th percentile.

For attributable mortality, 21 definitions (61.8%) described heat without separating moderate and extreme contributions. Temperatures above the MMT were the most frequent exposure range, accounting for 11 definitions (32.4% of all attributable mortality definitions). Other definitions covered temperatures above a selected percentile or absolute temperature, bounded temperature ranges or a specified calendar period. Six definitions (17.6%) described moderate heat, generally between the MMT and a high percentile, although one extended from the 50th to the 97.5th percentile. Seven definitions (20.6%) described extreme heat, most frequently above the 97th or 97.5th percentile, with others applying the 95th or 99th percentile.

### Part 2. Case Study

#### Minimum Mortality Temperature Approach

The MMT was identified at 20 °C, corresponding to the lowest predicted mortality (1,010 deaths) on the cumulative exposure–response curve. For overall heat exposure, the relative risk (RR) of mortality at the maximum temperature of 28.8 °C compared to the MMT was 1.48 (95% CI = [1.35, 1.63]). From this association, the corresponding attributable mortality during June-August 2022 was 20,313 deaths (95% CI = [16,244, 24,101]) (Table 4).

**Table 4:** Mortality risks and attributable mortality under the MMT and percentile-based approaches in Spain

Approach/ Heat definition	Temperature range	RR (95% CI)	Attributable deaths (95% CI)^c^
**MMT approach** ^**a**^			
Overall heat	> 20 °C	1.48 (1.35; 1.63)	20,313 (16,244; 24,101)
Moderate heat	20–26.6 °C	1.27 (1.20; 1.34)	8,651 (6,453; 10,771)
Extreme heat	> 26.6 °C	1.17 (1.09; 1.25)	11,662 (9,800; 15,014)
**Percentile-based ** ^**b**^			
Excessive heat	> 26.6 °C	1.17 (1.09; 1.25)	3,220 (1,520; 3,831)

^a^ Reference temperature at the MMT (20 °C).

^b^ Reference temperature at the 97.5th percentile (26.6 °C).

^c^ June-August 2022.

We defined moderate and extreme heat using the 97.5th percentile of the annual temperature distribution at 26.6 °C. Moderate heat comprised temperatures between the MMT and the 97.5th percentile, while extreme heat included temperatures above this threshold. For moderate heat, the RR comparing the 97.5th percentile with the MMT was 1.27 (95% CI = [1.20, 1.34]), and the attributable mortality across this range was 8,651 deaths (95% CI = [6,453, 10,771]). Extreme heat was defined as temperatures above the 97.5th percentile. The RR, calculated indirectly as the ratio of the RRs for overall and moderate heat, was 1.17 (95% CI = [1.09, 1.25]). Similarly, we estimated the attributable mortality by subtracting moderate heat deaths from overall heat deaths, yielding 11,662 deaths (95% CI = [9,800, 15,014]).

#### Percentile-Based Approach

The percentile-based approach defined excessive heat exposure as temperatures above the 97.5th percentile of the annual temperature distribution and centred the temperature–mortality exposure–response curve at this percentile. The RR comparing the maximum temperature with this threshold was 1.17 (95% CI = [1.09, 1.25]). Note that this mortality risk represents the same contrast as that for extreme heat under the MMT approach above, but it is estimated directly with the 97.5th percentile as the reference temperature for the comparison. The estimated excessive heat mortality above the 97.5th percentile was 3,220 deaths (95% CI = [1,520, 3,831]). This was approximately one-quarter of the extreme heat mortality estimated when using the MMT as the reference temperature.

However, both estimates concerned the same days and population and were derived from the same temperature–mortality exposure–response curve, but centred at two different reference temperatures. The difference therefore did not reflect a change in exposure or susceptibility, but different assumptions about baseline mortality risk. The MMT approach quantified all mortality attributable to extreme heat days relative to the lowest estimated mortality risk. Centring the curve at the 97.5th percentile restricted the estimate to deaths associated with mortality risk exceeding the risk estimated at the heat threshold. These deaths could therefore potentially be prevented if the protective measures activated at the 97.5th percentile were fully effective. The difference between the two estimates represents the heat-related deaths that would remain despite these preventive measures.

## Discussion

The increasing frequency and intensity of heat events underscore the importance of clear heat definitions for both climate change research and heat disaster preparedness [9, 10]. Our rapid review indicates that published studies used different approaches to translate the temperature–mortality exposure–response curve into a practical definition of heat. Studies make different choices about the heat thresholds and reference temperatures that together define the heat exposure contrast, including whether to separate moderate from extreme heat. These choices affect interpretation because they target different parts of the temperature–mortality exposure–response curve and can yield risk estimates that are not directly comparable across studies, even when similar modelling frameworks are used. Evidence from the literature also shows that alternative heat definitions can change estimated health associations, reinforcing the need for transparent and harmonised definition strategies [20]. Differences in data sources, analytical methods and reporting practices also complicate comparisons of heat-related mortality estimates between national systems [21]. As a result, risk communication becomes more difficult because the same terms can refer to different thresholds and different contrasts. This can confuse the general population and contribute to inconsistent messages in the media and among policymakers, a challenge explicitly recognised in guidance for heat health warning systems and heat health action plans [22]. Therefore, there is a need for a harmonised curve–based framework that explicitly specifies how heat is defined to improve comparability and facilitate translation into public health preparedness.

We propose a harmonisation framework for defining heat exposure that links the definition directly to the temperature–mortality exposure–response curve and clarifies the reference temperature used to express risks and burdens. The framework connects the research question and the intended application of the results with two analytical choices: the threshold delimiting heat exposure and the reference temperature defining the counterfactual. Together, these choices define the contrast and determine the interpretation of mortality risks and attributable burdens. These choices should be distinguished because restricting the calculation to days above a heat threshold does not necessarily mean taking that threshold as the reference temperature. To improve interpretability and comparability, future studies should report a minimal set of definition elements alongside the model specification. These include the reference temperature used to centre the curve, the threshold value used to define heat and, where applicable, moderate and extreme heat, the scale used to express thresholds (absolute temperature or percentile), and the lag window over which cumulative effects are computed. Although these elements are often reported in DLNM studies, their implications for the comparison being made should also be discussed [23].

The choice of reference temperature determines the counterfactual against which the mortality burden is estimated. Gasparrini and Leone [13] already stated that attributable measures can be calculated against alternative reference exposures, allowing different comparisons within the same modelling framework. Empirical studies also demonstrate the implications of these choices. Longden [24] showed that the reference temperature substantially affected estimates of heat- and cold-related mortality in Australia, while Liu et al. [25] reported substantial differences in heat-attributable disease burden when alternative minimum-risk exposure references were adopted. These findings support explicit justification of the reference temperature and interpretation of each estimate according to its counterfactual, rather than treating different estimates as interchangeable measures of the same burden.

In practice, the choice between the MMT approach and the percentile-based approach should depend on the research question and the intended application of the results. The MMT approach is useful for quantifying the overall heat-related burden, while the percentile-based approach may be better aligned with the operational logic of heat health warning systems, when the reference temperature corresponds to the activation threshold. It then quantifies the additional burden above the risk estimated at that point [26], which could be described as excessive heat mortality. These approaches are complementary because they address different epidemiological and public health questions. Although the percentile-based approach can align the burden estimate with an activation threshold, it does not determine where that threshold should be set. Benmarhnia et al. [27] found that lowering the activation threshold of New York City’s heat emergency plan was associated with fewer heat-related illnesses among older adults. The choice of activation point must also consider exposure duration, response capacity and the balance between broader protection and possible messaging fatigue [28]. Epidemiological risk thresholds therefore do not necessarily correspond to optimal intervention thresholds. The choice of heat thresholds and reference temperatures also depends on the geographical and population context. Differences in local climatology, acclimatisation, adaptation and vulnerability can justify different thresholds and reference temperatures [25]. Such variation is compatible with harmonisation, which concerns a common approach to defining and reporting heat exposure rather than uniform numerical values across settings.

However, we should acknowledge several limitations. The rapid review was restricted to PubMed and required temperature-related and mortality terms in article titles, potentially excluding relevant studies. Additional databases could identify further studies and change the reported frequencies of the approaches, but the heterogeneity in heat threshold percentiles and reference temperatures already documented would remain. The case study relies on national-level data from Spain and burden estimates for a single summer, limiting generalisability and concealing geographical and population differences. Nevertheless, keeping the data and model constant clarifies how changing the reference temperature alters both the estimated mortality burden and its meaning for epidemiological assessment and public health planning.

## Conclusion

Clear interpretation of heat effects is essential for effective risk communication with the public, social media, and policymakers. The term minimum risk may be misunderstood as implying no risk, and moderate heat may be interpreted as clinically unimportant even though large burdens can arise from temperatures that are common rather than extreme. Communicating these distinctions, with explicit heat thresholds and reference temperatures, can improve public understanding and support timely protective actions. Although our case study focuses on heat, the same principles could be extended to cold, strengthening the usefulness of curve-based definitions for year-round climate health risk communication and preparedness.

## Key References

- Armstrong B: Models for the relationship between ambient temperature and daily mortality. Epidemiology. 2006;17:624-631.
  - A foundational methodological paper on temperature–mortality modelling, providing the conceptual basis for later developments in time-series analyses of heat effects.
- Gasparrini A, Armstrong B, Kenward MG: Distributed lag non-linear models. Stat Med. 2010;29:2224-2234.
  - The methodological reference for distributed lag non-linear models, which have become the standard framework for modelling delayed and non-linear temperature–mortality associations.
- Gasparrini A, Guo Y, Hashizume M, Lavigne E, Zanobetti A, Schwartz J, Tobias A, Tong S, Rocklov J, Forsberg B, et al.: Mortality risk attributable to high and low ambient temperature: a multicountry observational study. Lancet. 2015;386:369-375.
  - A landmark multicountry study quantifying the mortality burden attributable to non-optimal temperatures, and a key reference for placing heat-related mortality in a global epidemiological context.
- Ebi KL, Vanos J, Baldwin JW, Bell JE, Hondula DM, Errett NA, Hayes K, Reid CE, Saha S, Spector J, et al.: Extreme Weather and Climate Change: Population Health and Health System Implications. Annu Rev Public Health. 2021;42:293-315.
  - A broad review that frames extreme heat within climate change and public health, emphasising implications for health systems, preparedness, and adaptation.

## Supplementary Information

Below is the link to the electronic supplementary material.

Supplementary File 1 (DOCX 223 KB)

## Data Availability

Daily mortality and temperature data analysed in the case study are publicly available from the Spanish Daily Mortality Monitoring System (MoMo) and the Spanish State Meteorological Agency (AEMET) OpenData, respectively.

## Code Availability

The R script is available from the first author upon reasonable request.
