# Supplementary material for: Heat Exposure Definitions in Temperature–Mortality Studies: Towards a Harmonisation Framework
Source: Curr Environ Health Rep. 2026 Oct 23;13(1):49. doi: 10.1007/s40572-026-00571-5 (PMC13694548; doi:10.1007/s40572-026-00571-5)
Supplement: Supplementary file 1 [file 40572_2026_571_MOESM1_ESM.docx]

**HEAT EXPOSURE THRESHOLDS IN TEMPERATURE–MORTALITY STUDIES: TOWARDS A HARMONISATION FRAMEWORK**

**Supplementary Material**

Appendix. PRISMA 2020 checklist ……………………………………………………………..… 2

Supplementary Table 1. Search strategy for search in Pubmed ………………………… 5

Supplementary Figure 1. Flowchart of assessment of eligible studies ………………. 6

Supplementary Table 2. Description of the studies included ……………………………. 7

References for studies included ………………………...……….……………….……………. 13

**Appendix.** PRISMA 2020 checklist.

The reporting items below are adapted to the rapid review’s objective of characterising heat exposure definitions rather than synthesising temperature–mortality effect estimates. This is a study-specific checklist, not an official PRISMA-RR extension. Page numbers refer to the manuscript unless explicitly identified as protocol pages.

| **Section / Topic** | **Item** | **Reporting item adapted to this methodological rapid review** | **Page and section** |
| --- | --- | --- | --- |
| **TITLE** |  |  |  |
| Title | 1 | Identify the report as a rapid review of methodological definitions. | Page 2. Abstract; Page 4. Introduction; Page 6 Methods, Part1. |
| **ABSTRACT** |  |  |  |
| Abstract | 2 | Summarise the methodological objective, review methods, principal findings and implications. | Page 2. Abstract. |
| **INTRODUCTION** |  |  |  |
| Rationale | 3 | Explain why variation in heat exposure definitions warrants review and how it affects interpretation and comparability. | Pages 3–4. Introduction. |
| Objectives | 4 | State the questions concerning heat thresholds, reference temperatures and their implications for mortality estimates. | Page 4. Introduction. |
| **METHODS** |  |  |  |
| Eligibility criteria | 5 | Specify eligible study designs, populations, mortality outcomes, exposure metrics, analytical methods, publication types, dates and languages. | Page 6. Methods, Part 1, Search strategy. |
| Information sources | 6 | Identify the sources searched and distinguish the publication cut-off from the date the search was performed. | Page 6. Methods, Part 1, Search strategy. |
| Search strategy | 7 | Present the full search strategy, including title restrictions, filters and other limits. | Page 6. Methods, Part 1, Search strategy; Supplementary Table 1. |
| Selection process | 8 | Describe screening and full-text eligibility assessment, reviewer independence and resolution of disagreements. | Page 6. Methods, Part 1, Study selection and data extraction. |
| Data collection process | 9 | Describe extraction procedures, reviewer independence, resolution of disagreements and management of records and extracted data. | Pages 6–7. Methods, Part 1, Study selection and data extraction. Protocol, page 3, Section 3.8. |
| Methodological data items | 10a | Specify the heat thresholds and reference temperatures extracted, including definitions of overall, moderate and extreme heat and their application to mortality risk or attributable mortality. Describe the handling of multiple definitions within a study. | Pages 5–7. Methods, Conceptual framework; Part 1, Study selection and data extraction. |
| Other data items | 10b | Specify the study characteristics extracted, including setting, period, design, mortality outcomes, temperature metrics and lag windows. Describe the handling of missing or unclear information. | Pages 6–7. Methods, Part 1, Study selection and data extraction. |
| Assessment of study limitations | 11 | State whether study risk of bias was assessed and explain any omission in relation to the methodological objective. Distinguish this from assessment of reporting transparency. | Page 7. Methods, Part 1, Synthesis approach. Protocol, page 3, Section 3.9. |
| Mortality effect measures | 12 | Distinguish definitions reported for mortality risk from those reported for attributable mortality. Clarify that the definitions, rather than the effect estimates themselves, are synthesised. | Pages 7 and 9. Methods, Part 1, Synthesis approach; Results, Part 1, Heat exposure definitions. |
| Grouping for synthesis | 13a | Describe how studies and definitions were grouped according to reference temperatures, heat values, heat categories and mortality effect measures. | Page 7. Methods, Part 1, Synthesis approach. |
| Preparation of extracted data | 13b | Describe any terminology standardisation, classification rules, conversions or treatment of incomplete information used to prepare the extracted definitions. | Page 7. Methods, Part 1, Synthesis approach. |
| Tabulation and presentation | 13c | Describe how individual study characteristics and methodological definitions were organised into tables or figures. | Page 7. Methods, Part 1, Synthesis approach. Protocol, page 4, Section 3.12. |
| Narrative and descriptive synthesis | 13d | Describe the narrative synthesis and frequency summaries, distinguishing studies from individual definitions and specifying the denominators used. | Pages 7 and 9–10. Methods, Part 1, Synthesis approach; Results, Part 1, Heat exposure definitions. |
| Investigation of variation | 13e | Describe any formal analyses investigating why definitions differed across settings, populations or study characteristics. | Not applicable to the descriptive methodological synthesis undertaken in this review |
| Sensitivity analyses | 13f | Describe any sensitivity analyses assessing the robustness of the methodological synthesis or classification. | Not applicable to the descriptive methodological synthesis undertaken in this review |
| Reporting bias assessment | 14 | State whether publication bias or selective reporting was assessed and explain any omission in relation to the review objective. | Protocol, page 3, Section 3.9. |
| Certainty assessment | 15 | State whether certainty of evidence was assessed and explain why such assessment was or was not relevant to the methodological synthesis. | Page 7. Methods, Part 1, Synthesis approach. Protocol, page 4, Section 3.11. |
| **RESULTS** |  |  |  |
| Selection results | 16a | Report the numbers identified, screened, assessed for eligibility and included, preferably in a flow diagram. | Page 9. Results, Part 1, Study selection and characteristics; Supplementary Figure 1. |
| Full-text exclusions | 16b | Identify potentially eligible reports excluded after full-text assessment and provide the reasons for exclusion. | Page 9. Results, Part 1, Study selection and characteristics; Supplementary Figure 1. |
| Study characteristics | 17 | Identify the included studies and present their settings, designs, periods, mortality outcomes, temperature metrics and lag windows. | Page 9. Results, Part 1, Study selection and characteristics; page 17. Table 1; Supplementary Table 2. |
| Assessment findings | 18 | Present study-level risk of bias assessments if undertaken, distinguishing them from findings about reporting transparency. | Not applicable to the descriptive methodological synthesis undertaken in this review. |
| Individual methodological findings | 19 | Present the reference temperatures and heat exposure definitions extracted from each study, distinguishing mortality risk and attributable mortality where applicable. | Supplementary Table 2. |
| Characteristics of the evidence base | 20a | Summarise the characteristics of the studies contributing to the methodological synthesis. | Page 9. Results, Part 1, Study selection and characteristics; page 17. Table 1. |
| Descriptive synthesis findings | 20b | Present the frequencies of reference temperatures and heat exposure definitions, identifying whether counts represent studies or definitions. | Pages 9–10. Results, Part 1, Reference temperatures; Heat exposure definitions; pages 18–19. Tables 2–3. |
| Explanations for variation | 20c | Present results of any formal analyses investigating reasons for differences in definitions. | Not applicable to the descriptive methodological synthesis undertaken in this review |
| Sensitivity findings | 20d | Present results of any sensitivity analyses of the methodological synthesis. | Not applicable to the descriptive methodological synthesis undertaken in this review |
| Reporting bias findings | 21 | Present reporting bias assessments if undertaken. | Not applicable to the descriptive methodological synthesis undertaken in this review. |
| Certainty findings | 22 | Present certainty assessments if undertaken. | Not applicable to the descriptive methodological synthesis undertaken in this review. |
| **DISCUSSION** |  |  |  |
| Interpretation | 23a | Interpret variation in heat thresholds and reference temperatures in relation to previous methodological and empirical evidence. | Pages 12–14. Discussion. |
| Limitations of the reviewed evidence | 23b | Discuss limitations arising from the definitions and reporting available in the included studies. | Pages 12–13. Discussion. |
| Limitations of review procedures | 23c | Discuss how search restrictions and abbreviated or omitted procedures may affect completeness and the observed distribution of methodological practices. | Page 14. Discussion, limitations paragraph. |
| Implications | 23d | Explain implications for defining and reporting heat exposure, interpreting mortality estimates and applying findings to public health. | Pages 12–14. Discussion; page 14. Conclusion. |

| **OTHER INFORMATION** |  |  |  |
| --- | --- | --- | --- |
| Registration | 24a | Provide the registration platform and identifier for the rapid review protocol. | Page 6. Methods, Part 1, Search strategy. |
| Protocol access | 24b | Indicate where the protocol is accessible. | Page 6. Methods, Part 1, Search strategy. Protocol, page 5, Section 8. |
| Protocol amendments | 24c | Report and explain changes from the protocol, distinguishing planned procedures from those actually implemented. | Protocol, page 4, Section 7. |
| Support | 25 | Describe review-specific support and the role of funders, distinguishing these from individual author funding. | Page 16. Acknowledgements. Protocol, page 4, Section 6. |
| Competing interests | 26 | Declare competing interests of the review authors. | Page 16. Declarations, Conflict of Interest. |
| Availability of review materials | 27 | State where extracted study data, extraction forms, classification materials and review analysis code can be accessed. | Supplementary Table 2. |
| **ADDITIONAL RAPID REVIEW ITEMS [1]** |  |  |  |
| Prespecified iterative methods | RR1 | State whether decisions about scope or methods during the review followed an iterative process specified in advance. Describe any decisions and when they occurred. | Not reported. |
| Abbreviated or omitted procedures | RR2 | Explain the specific restrictions and omissions that make this a rapid review, including the focused search and assessments not undertaken. | Pages 6–7. Methods, Part 1, Search strategy; Synthesis approach; page 14. Discussion. Protocol, Sections 3.6, 3.9, 3.11 and 4. |
| Knowledge user involvement | RR3 | State whether policymakers, practitioners, patients or other intended users contributed to the review and describe their involvement. | No knowledge users were involved in the review. |
| Authorship and correspondence | RR4 | Identify the authors, their contributions and the corresponding author’s contact details. | Page 1. Title page; page 16. Author Contribution. |
| Acknowledgements | RR5 | Identify non-author contributions to the development, conduct or reporting of the review. | Page 16. Acknowledgements. |
| Peer review | RR6 | Describe peer review during preparation of the report, including whether reviewers were internal or external and their relevant expertise. | Page 16. Acknowledgements. |

**Reference**

1. Stevens A, Hersi M, Garritty C, Hartling L, Shea BJ, Stewart LA, Welch VA, Tricco AC: Rapid review method series: interim guidance for the reporting of rapid reviews. BMJ Evid Based Med 2025, 30:118-123.

**Supplementary Table 1.** Search strategy for Pubmed.

(dlnm OR "distributed lag non-linear model*" OR "distributed lag nonlinear model*") AND (time-series OR "time series" OR case-crossover) AND ( ((high[ti] OR hot[ti] OR extreme[ti] OR nonoptimal[ti] OR non-optimal[ti]) AND temperature*[ti]) OR heat[ti] ) AND mortality[ti]

**Supplementary Figure 1.** Flowchart of assessment of eligible studies.

Duplicate records removed (n = 0)

Records identified from:

Databases (n = 1)

Registers (n = 78)

**Identification**

Records screened

(n = 78)

Records excluded

(n = 23)

Reports sought for retrieval

(n = 55)

Reports not retrieved

(n = 0)

**Screening**

Reports assessed for eligibility

(n = 55)

Reports excluded:

Did not use daily temperature (n = 8)

Focused in other stressors (n = 5)

Not conducted in humans	(n = 1)

Studies included in review

(n = 41)

**Included**

**Supplementary Table 2.** Description of the studies included.

| **Study, year [ref]** | **Location/s** | **Country** | **Period** | **Mortality outcome/s (ICD)** | **Study design** | **Temperature metric/s** | **Lags** |
| --- | --- | --- | --- | --- | --- | --- | --- |
| Lin et al., 2012 [1] | 3 metropolitan areas comprising 6 cities/counties | Taiwan | 2000-2008 | All-cause (ICD9:0-999) | Time-series with GAM | Mean, maximum, heat index, humidex,  temperature humidity index, apparent temperature,  wet-bulb globe temperature | 7 days |
| Chen et al., 2013 [2] | 8 cities | China | 1996-2008 | Stroke (ICD-10: I60–I69) | Time-series with GAM | Mean apparent temperature | 14 days |
| Ma et al., 2014 [3] | 17 cities | China | 1996-2008 | Non-accidental (ICD-10: A00–R99),  cardiovascular (ICD-10: I00–I99),  respiratory (ICD-10: J00–J98) | Time-series with GAM | Mean | 14 days |
| Ng et al., 2014 [4] | 6 cities | Japan | 2002-2007 | Non-accidental (ICD10:A00-R99) | Time-series | Mean | 20 days, 0, 0–1 days (heat) |
| Petkova et al., 2014 [5] | New York City | USA | 1900-1948,  1973-2006 | All-cause | Time-series | Mean | 5 days |
| de'Donato et al., 2015 [6] | 9 cities | Finland, France, Greece, Hungary, Italy, Spain, Sweden, UK | 1996–20010 | Non-accidental (ICD-9: 1–799),  cardiovascular (ICD-9: 390–459), respiratory (ICD-9: 460–519) | Time-series | Mean, maximum, minimum apparent temperature | 1 to 5 days |
| Gasparrini et al., 2015 [7] | 384 locations | Australia, Brazil, Canada, China, Italy, Japan, South Korea, Spain, Sweden, Taiwan, Thailand, UK, USA | 1985-2012 | All cause, Non-accidental (ICD10:A00-R99, ICD9:0-799) | Time-series | Mean | 21 days |
| Lubczynska et al., 2015 [8] | Cyprus | Cyprus | 2004-2010 | Hypertensive diseases (ICD10:I10-I13),  ischemic heart disease (ICD10:I20-I25),  other heart diseases (ICD10:I26-I51),  cerebrovascular (ICD10:I60-I69),  other circulatory system (ICD10:I71-I99) | Case-crossover | Mean, maximum, minimum | 10 days |
| Qiao et al., 2015 [9] | Brisbane | Australia | 1996–2004 | Non-accidental (ICD10:A00-R99, ICD9:1-799), cardiovascular (ICD10:I00-I99, ICD9:390-459),  respiratory (ICD10:J00-J99, ICD9:460-519) | Time-series with GAM | Mean | 0–21 days |
| Cui et al., 2016 [10] | Chengdu | China | 2011-2014 | Non-accidental,  cardiovascular, respiratory | Time-series | Mean | 21 days |
| Achebak et al., 2018 [11] | 47 cities | Spain | 1980-2015 | Cardiovascular (ICD10:I00-I99, ICD9: 390-459), respiratory (ICD10: J00-J99, ICD9: 460-519) | Time-series | Mean | 10 days |
| Aboubakri et al., 2019 [12] | Kerman | Iran | 2005–2017 | All-cause | Time-series | Mean, maximum, minimum | 14 days |
| Deng et al., 2019 [13] | Yuxi | China | 2009–2016 | Non-accidental (ICD10:A00-R99), cardiovascular (ICD10:I00-I99),  heart (ICD10:I00-I51),  stroke (ICD10:I60-I69),  respiratory (ICD10:J00-J99) | Time-series | Mean | 28 days |
| Luan et al., 2019 [14] | 31 capital cities | China | 2008-2013 | Suicide (ICD10:X60-X84) | Time-series | Mean | 7 days |
| Alahmad et al., 2020 [15] | Kuwait | Kuwait | 2010-2016 | Non-accidental (ICD10:A00-R99),  cardiovascular (ICD10:I00-I99) | Time-series | Mean | 30 days |
| Ellena et al., 2020 [16] | Turin | Italy | 1982-2018 | All-cause | Time-series | Mean | 7 days |
| Sera et al., 2020 [17] | 331 locations | Canada, Japan, Spain, USA | 1972–2009 | All-cause,  non-accidental (ICD10:A00-R99, ICD9:0-799) | Time-series | Mean | 2 days |
| Saucy et al., 2021 [18] | Zurich | Switzerland | 2000–2015 | Cardiovascular (ICD10:I00-I99), ischemic heart disease (ICD10:I20–I25),  myocardial infarction (ICD10:I21–I22),  stroke (ICD10:I60–I64),  heart failure (ICD10:I50), hypertensive diseases (ICD10:I10–I15), arrhythmia (ICD10: I44–I49) | Case-crossover | Mean, maximum, minimum | 0–14 days,  heat 7 days |
| Casas et al., 2022 [19] | Brussels | Belgium | 2002-2011 | Suicide (ICD10:X60-X84, Y10-Y34) | Case-crossover | Mean | 6 days |
| de Schrijver, 2022 [20] | 2,056 municipal units | Switzerland | 1969-2017 | All-cause | Case time-series | Mean | 21 days |
| Ellena et al., 2022 [21] | Turin | Italy | 1982-2018 | All-cause | Time-series | Mean | 21 days |
| Ingole et al., 2022 [22] | Pune | India | 2004-2012 | All-cause | Case-crossover | Mean | 21 days |
| Lee et al., 2022 [23] | 229 districts | South Korea | 2011-2017 | All-cause,  cardiovascular (ICD-10:I00–I99),  respiratory (ICD-10:J00–J99),  other causes | Time-series | Mean | 10 days |
| Roca-Barcelo et al., 2022 [24] | Sao Paulo | Brazil | 2000-2018 | Non-external (ICD-10:A00–R99),  cardiovascular (I00–I99),  respiratory (J00–J99) | Time-series | Mean | 21 days |
| Achebak et al., 2023 [25] | 48 provinces | Spain | 1980–2018 | All-cause | Time-series | Mean | 10 days |
| Quilty et al., 2023 [26] | Northern Territory (163 locations) | Australia | 1980-2019 | All-cause | Case time-series | Mean | 3 days |
| Ragettli et al., 2023 [27] | 7 regions | Switzerland | 2003-2016 | Non-accidental (ICD10:A00–R99) | Case-crossover | Mean, maximum, minimum | 7 days |
| Shrikhande et al., 2023 [28] | Puducherry | India | 2011-2020 | In-hospital cardiovascular mortality,  ischaemic heart disease,  cerebrovascular accidents  other cardiovascular | Case-crossover | Apparent temperature | 21 days |
| Tobias et al., 2023 [29] | 52 cities | Spain | 1990-2016 | Heatstroke (ICD10:X30, ICD9:992) | Case-crossover | Mean | 14 days |
| Alahmad et al., 2024 [30] | 522 cities | Brazil, Canada, Costa Rica, Cyprus, Ecuador, Estonia, Finland, Guatemala, Italy, Japan, Kuwait, Moldova, Panama, Paraguay, Philippines, Portugal, South Africa, Spain, Switzerland, Taiwan, Thailand, UK, USA, Uruguay, Vietnam | 1979-2019 | Ischemic stroke (ICD10:I63-I66, ICD9:433-434), hemorrhagic stroke (ICD10:I60-I62, ICD-9:430-432) | Case-crossover | Mean | 14 days |
| Alwadi et al., 2024 [31] | Amman | Jordan | 2000–2018 | All-cause | Time-series | Mean | 10 days |
| Psistaki et al., 2024 [32] | Athens, Thessaloniki, Cyprus | Greece, Cyprus | 1999–2019 | Cardiovascular (ICD10:I00-I99) | Time-series | Mean | 21 days |
| Ragettli et al., 2024 [33] | All municipalities | Switzerland | 2003-2016 | All-cause (ICD10:A00–Z99), neoplasms (ICD10:C00-D48), endocrine diseases (ICD10:E00-E90),  mental and behavioural disorders (ICD10:F00-F99), nervous system (ICD10:G00-G99),  cardiovascular (ICD10:I00-I99),  respiratory (ICD10:J00-J99),  digestive (ICD10:K00-K93),  renal (ICD10:N00-N39),  external causes (ICD10:V01-Y98),  cerebrovascular (ICD10:I60-I69),  stroke (ICD10:I60-I64),  ischaemic heart disease (ICD10:I20-I25),  myocardial infarction (ICD10:I21-I22),  hypertensive diseases (ICD10:I10-I15),  diabetes (ICD10:E10-E14),  Alzheimer/dementia (ICD10:F00-F03,G30-G31.1,G31.8-G31.9), chronic obstructive pulmonary disease (ICD10:J41-J44),  suicide (ICD10:X60-X84),  accidents (ICD10:V01-X59) | Case-crossover | Maximum, minimum | 7 days |
| Wicki et al., 2024 [34] | 8 cities | Switzerland | 2003-2016 | Non-accidental (ICD10:A00-R99) | Case time-series | Maximum temperature, binary warm-night indicator | 3 days |
| Yi et al., 2024 [35] | 13 cities | China | 2016-2019 | All-cause | Time-series | Maximum | 21 days |
| Ali et al., 2025 [36] | Flanders | Belgium | 2018-2021 | All-cause | Case-crossover | Maximum, minimum | 14 days |
| Chu et al., 2025 [37] | Contiguous USA (3,108 counties) | USA | 2000-2020 | All-cause, endocrine, nutritional and metabolic (ICD-10: E00–E89), mental, behavioral and neurodevelopmental (F01–F99), nervous system (G00–G99), cardiovascular (I00–I99), respiratory (J00–J99), digestive diseases (K00–K95), external causes (V00–Y99) | Case-crossover | Mean, maximum, minimum | 6 days |
| Frasch et al., 2025 [38] | 401 counties | Germany | 2010-2019 | In-hospital deaths | Time-series | Mean | 28 days |
| Lu et al., 2025 [39] | Mainland China (2,844 county-level administrative units) | China | 2013-2019 | Diabetes (ICD-10: E10–E14) | Case-crossover | Mean | 7 days |
| Sieck et al., 2025 [40] | Contiguous USA | USA | 2012-2018 | Patients with end-stage kidney disease | Case-crossover | UTCI | 3 days |
| Zhang et al., 2025 [41] | Wuhu | China | 2014-2020 | Cardiovascular (ICD10:I00-I99),  ischemic heart disease (ICD10:I20-I25) stroke (ICD10:I60-I69) | Time-series with GAM | DMT, DTR | 21 days |

**Supplementary Table 2.** Description of the studies included (continuation).

| **Study, year [ref]** | **Extracted text (section)** | **Reference temperatures** | **Heat exposure definitions** |
| --- | --- | --- | --- |
| Lin et al., 2012 [1] | Cumulative 8-day relative risks were estimated at 1 and 2 standard deviations above the mean, corresponding to the 84.1st and 97.7th percentiles, respectively, using the Z scores associated with the lowest risks as reference values. (Abstract, Methods) The reference values were Z=0 in Taipei and Central Taiwan, and Z=−1 in Southern Taiwan. (Table 3, footnote) | MMT (Z=0 or Z=−1) | Mortality risk: at the 84.1^st^ and 97.7^th^ percentiles (Z=+1 and Z=+2) |
| Chen et al., 2013 [2] | Extreme heat was defined at the 99th percentile of temperature and heat at the 90th percentile, both compared with the 75th percentile over lags 0–3 days. (Figure 3, caption) The full temperature range was expressed as relative risks over lags 0–14 days with the MMT as the reference temperature. (Figure 2, caption) | 75^th^ percentile | Mortality risk: heat at the 90^th^ percentile, extreme heat at the 99^th^ percentile. |
| Ma et al., 2014 [3] | For heat, we calculated the relative risks comparing the 99th and 75th percentiles of temperature in each city. (§2.2 Statistical analysis) The full temperature range was expressed as relative risks over lags 0–14 days with the MMT as the reference temperature. (Figure 1, caption) | 75^th^ percentile | Mortality risk: at the 99^th^ percentile. |
| Ng et al., 2014 [4] | For heat, mortality risk was compared between the maximum temperature and the 80th percentile of temperature in each city. (Statistical analysis) To compute the relative effect of heat, we compared mortality risk at the 99th and 90th percentiles of the city-specific temperature distribution. (Statistical analysis) To determine the absolute effect of heat, we compared mortality risk at 29 °C (the pooled 95th percentile) with that at 23.5 °C (the pooled 80th percentile). (Statistical analysis) | 90^th^ percentile | Mortality risk: at the 99^th^ percentile. |
| Petkova et al., 2014 [5] | We developed the decadal models using daily mean temperature, with 22 °C (approximately the 80th percentile of annual temperature) as the reference temperature. (Analysis of Temperature-Mortality Relations) The decadal cumulative relative risk at 29 °C (approximately the 99th percentile of daily mean temperature) summarised the heat effect. (Analysis of Temperature-Mortality Relations) | 22°C | Mortality risk: at 29°C |
| de'Donato et al., 2015 [6] | The effect of high temperatures on mortality was expressed as relative risks comparing the 99th with the 75th percentile of the period-specific summer temperature distribution. (§2.4 Statistical Analysis) The total number of attributable deaths was calculated by summing the daily attributable deaths for temperatures between the 75th and 99th percentiles. (§2.4 Statistical Analysis) | 75^th^ percentile | Mortality risk: at the 99^th^ percentile.  Attributable mortality: between 75^th^ -99^th^ percentiles. |
| Gasparrini et al., 2015 [7] | We calculated cold- and heat-attributable mortality by summing daily contributions at temperatures below and above the MMT, respectively. Extreme cold and heat were defined as temperatures below the location-specific 2.5th percentile and above the location-specific 97.5th percentile, respectively. Moderate cold and heat were defined as the intervals between the MMT and these thresholds. We defined other ranges using thresholds at the 10th, 25th, 50th, 75th and 90th percentiles. (Methods) | MMT (location-specific) | Attributable mortality: overall heat >MMT, moderate heat: MMT–97.5^th^ percentile, extreme heat >97.5^th^ percentile. |
| Lubczynska et al., 2015 [8] | The median temperatures were selected as the reference temperatures for daily maximum, mean and minimum temperature, corresponding to 25.9 °C, 20.0 °C and 14.1 °C, respectively. (Data analysis) Heat effects were estimated at the 90th, 95th and 99th percentiles and at the maximum observed temperature for each metric. (Data analysis) | 50^th^ percentile | Mortality risk: at the 90^th^, 95^th^, 99^th^ percentiles, and at the maximum temperature. |
| Qiao et al., 2015 [9] | A daily mean temperature of 28 °C was chosen as the common heat threshold over lags 0–1 for all mortality types. (Statistical methods) We estimated the relative risk of mortality at 29 °C compared with the 28 °C threshold. (Statistical methods) Effects were also expressed as the percentage increase in daily mortality per 1 °C increase above 28 °C, over lags 0–1. (Table 3) | 28°C | Mortality risk: at 29°C |
| Cui et al., 2016 [10] | Heat and cold were defined as temperatures above and below the MMT, respectively. (Abstract) Extreme cold and heat were defined as temperatures below the location-specific 2.5th percentile and above the location-specific 97.5th percentile, respectively. Moderate cold was defined between the 2.5th percentile and the MMT, and moderate heat between the MMT and the 97.5th percentile. (§2.2.3 Attributable components) | MMT (Cause, sex, and age-specific) | Attributable mortality: overall heat >MMT, moderate heat: MMT–97.5^th^ percentile, extreme heat >97.5^th^ percentile. |
| Achebak et al., 2018 [11] | We summarised the results by calculating the pooled relative risks at the 90th and 99th temperature percentiles from year-specific curves between 1980 and 2015. (Statistical analysis) Overall heat-attributable mortality was calculated by summing daily contributions at temperatures above the MMT. Moderate heat was defined between the city-specific MMT and the city-specific 97.5th percentile, and extreme heat above this threshold. (Statistical analysis) | MMT (city, cause, and sex-specific) | Mortality risk: at the 90^th^ and 99^th^ percentiles Attributable mortality: overall heat >MMT, moderate heat: MMT–97.5^th^ percentile, extreme heat >97.5^th^ percentile. |
| Aboubakri et al., 2019 [12] | Temperatures above the MMT were defined as heat. Mild heat was defined between the MMT and the 99th percentile, and extreme heat above the 99th percentile. (§2.3 Heat definition) The MMT was selected as the reference temperature. (§2.4 Modeling strategy) | MMT (22.7°C) | Attributable mortality: overall heat >MMT, moderate heat: MMT–99^th^ percentile, extreme heat >99^th^ percentile. |
| Deng et al., 2019 [13] | We calculated the total attributable fraction and separated cold- and heat-attributable mortality by summing daily contributions at temperatures below and above the median temperature, respectively. Extreme cold and heat were defined as temperatures below the 2.5th percentile (5.4 °C) and above the 97.5th percentile (23.1 °C) of daily mean temperature. Mild cold and heat were defined as the intervals between the median temperature and these thresholds. (Methods) | 50^th^ percentile | Attributable mortality: overall heat >50^th^ percentile, moderate heat: 50^th^ –97.5^th^ percentile, extreme heat >97.5^th^ percentile. |
| Luan et al., 2019 [14] | The MMT was selected as the reference temperature in each city. High-temperature relative risks were estimated at the 95th percentile. (Statistical analysis) | MMT (city-specific) | Mortality risk: at the 95^th^ percentile. |
| Alahmad et al., 2020 [15] | The heat effect was estimated by comparing mortality risk at the 99th percentile with that at the MMT. (Table 3) | MMT (34.7°C, 66^th^ pctile) | Mortality risk: at the 99^th^ percentile. |
| Ellena et al., 2020 [16] | Heat-related mortality risk was summarised by the relative risk at the 99th percentile of daily summer temperatures. Attributable fractions and numbers of deaths were calculated for total heat at temperatures above the MMT, moderate heat between the MMT and the 97th percentile, and extreme heat above the 97th percentile. (Statistical analysis) | MMT (sex, age and socioeconomic-specific) | Mortality risk: at the 99^th^ percentile. Attributable mortality: overall heat >MMT, moderate heat: MMT–97^th^ percentile, extreme heat >97^th^ percentile. |
| Sera et al., 2020 [17] | Results were reported as country-averaged relative risks, using the MMT as the reference temperature. (Methods) Heat-attributable deaths were summed for days above the location-specific 50th percentile of summer temperatures and divided by total deaths to calculate the attributable fraction. (Methods) Heat-related relative risks compared the 99th percentile with the MMT. (Results) | MMT (country-specific) | Mortality risk: at the 99^th^ percentile. Attributable mortality: >50^th^ percentile of the summer-season. |
| Saucy et al., 2021 [18] | We estimated mortality odds ratios comparing the 99th percentile of annual daily mean temperature with the optimum temperature of 20 °C for heat, and the 5th percentile with the same reference for cold. (§2.3 Statistical analysis) The 20 °C reference temperature was derived from an optimum temperature previously estimated for Switzerland and corresponded approximately to the 90th percentile in our data. (§2.3 Statistical analysis) The mortality fraction attributable to temperatures above 20 °C was 2%. (Results) | MMT (20°C) | Mortality risk: at the 99^th^ percentile. Attributable mortality: >20 °C |
| Casas et al., 2022 [19] | Relative risks were estimated at the 5th and 95th percentiles for moderate cold and heat, and at the 1st and 99th percentiles for extreme cold and heat, respectively, with the median as the reference temperature. (§2.3 Statistical analyses) | 50 ^th^ percentile | Mortality risk: moderate heat at the 95^th^ percentile, extreme heat at the 99^th^ percentile. |
| de Schrijver, 2022 [20] | We computed cold- and heat-related excess mortality by summing daily contributions at temperatures below and above the decade-age-canton–specific MMT, respectively. (Methods) | MMT (decade, age and canton-specific) | Attributable mortality: >MMT. |
| Ellena et al., 2022 [21] | The temperature–mortality relative risk curves were centred at the subperiod-specific MMT and used to compute temperature-attributable mortality throughout the year. (§2.2 Statistical analysis) | MMT (period-specific) | Mortality risk: at the 99^th^ percentile. Attributable mortality: between June–August. |
| Ingole et al., 2022 [22] | Cold- and heat-attributable mortality were calculated by summing daily contributions at temperatures below and above the MMT, respectively. (§2.3 Statistical analysis) Relative risks were reported at the 1st, 2.5th, 97.5th and 99th percentiles for extreme cold, moderate cold, moderate heat and extreme heat, respectively. (Results) | MMT (28.6°C, 85.1^st^ pctile) | Mortality risk: moderate heat at the 97.5^th^ percentile, extreme heat at the 99^th^ percentile. Attributable mortality: >MMT. |
| Lee et al., 2022 [23] | Heat-related mortality risk was estimated by comparing the 99th percentile of the summer temperature distribution across all districts with the MMT. (Statistical analyses) | MMT (district-specific) | Mortality risk: at the 99^th^ percentile of the summer-season. |
| Roca-Barcelo et al., 2022 [24] | We used changes in MMT and in the relative risks associated with extreme heat (99th percentile), moderate heat (90th percentile), moderate cold (10th percentile) and extreme cold (1st percentile) as indicators of adaptation. The year-specific MMT served as the reference temperature. (Methods) | MMT (year-specific) | Mortality risk: moderate heat at the 90^th^ percentile, extreme heat at the 99^th^ percentile. |
| Achebak et al., 2023 [25] | The exposure–response functions were centred at the MMT. Heat and cold days were defined as days with temperatures above and below the MMT, and extreme heat and cold days as those above the 95th and below the 5th temperature percentiles, respectively. (§2.2 Statistical analysis) | MMT (province-specific) | Mortality risk: at the 99^th^ percentile of summer temperature. Attributable mortality: overall heat >MMT, extreme heat: >95^th^ percentile. |
| Quilty et al., 2023 [26] | Hot weather was defined as a mean temperature over the 3-day lag period of ≥35 °C. (Statistical analysis) | Not reported | Heat: 3-day mean temperature ≥35°C |
| Ragettli et al., 2023 [27] | Mortality odds ratios compared potential heat-warning thresholds at the 90th, 92.5th, 95th, 98th, 99th and 99.5th percentiles of the warm-season temperature distribution with the median warm-season temperature. (§2.4.2 Threshold Temperatures) | 50^th^ percentile of warm-season | Mortality risk: at the 90^th^, 92.5^th^, 95^th^, 98^th^, 99^th^ and 99.5^th^ percentiles of warm-season. |
| Shrikhande et al., 2023 [28] | We expressed risks relative to the MMT. (Statistical model) Temperatures below and above 34 °C were defined as cold and heat, respectively. (Results) Lag-specific relative risks were reported at the 5th (27.3 °C) and 95th (38.0 °C) percentiles of apparent temperature over 21 days. (Results) | MMT (33.9°C, 60^th^ pctile) | Mortality risk: at the 95 percentile. Attributable mortality: >34 °C |
| Tobias et al., 2023 [29] | The overall relative risk across all lags was estimated per 1 °C increase above the 16 °C threshold. (Results) | 16°C | Mortality risk risk per 1°C increase. |
| Alahmad et al., 2024 [30] | We calculated cumulative relative risks across all lag days with the MMT as the reference temperature. Extreme heat and cold relative risks were reported at the 99th and 1st percentiles, respectively. For attributable mortality, extreme heat was defined as days with temperatures above the city-specific 97.5th percentile. (Statistical Analysis) | MMT (city-specific) | Mortality risk: at the 99^th^ percentile. Attributable mortality: >97.5^th^ percentile. |
| Alwadi et al., 2024 [31] | Heat- and cold-attributable mortality were estimated for days with temperatures above and below the MMT, respectively. (Results) | MMT (sex and nationality-specific) | Attributable mortality: >MMT. |
| Psistaki et al., 2024 [32] | Cold- and heat-attributable fractions were estimated for temperatures below and above the subperiod-specific MMT, respectively. (§2.2 Statistical analysis) Moderate heat was defined at the 90th percentile and extreme heat at the 99th percentile. (Table 1) | MMT (location and period-specific) | Mortality risk: moderate heat at the 90^th^ percentile, extreme heat at the 99^th^ percentile. Attributable mortality: >MMT |
| Ragettli et al., 2024 [33] | The associations were centred at the cause-specific MMT. Extreme heat effects were expressed as cumulative mortality odds ratios over lags 0–7, comparing the 98th percentile of warm-season daily maximum temperature (33 °C) with the cause-specific MMT. (Statistical analysis) | MMT (cause, region, and period-specific) | Mortality risk: at the 98^th^ percentile of warm-season. |
| Wicki et al., 2024 [34] | We expressed the results as cumulative relative risks across the full lag period, comparing the 99th percentile of warm-season daily maximum temperature (35 °C) with the MMT. Warm-night effects were estimated using a binary indicator comparing daily minimum temperatures ≥20 °C with those <20 °C. (§2.6 Statistical analysis) | MMT (22.5°C) | Mortality risk: at the 99^th^ percentile of warm-season. |
| Yi et al., 2024 [35] | We identified the MMT and estimated city-specific mortality relative risks comparing each heat-alert threshold, such as 35 °C, with the MMT. We estimated the numbers and fractions of deaths attributable to three temperature intervals, ≤35 °C, 35–37 °C and >37 °C. (Methods) | MMT (city-specific) | Mortality risk: at 35°C and 37°C Attributable mortality: ≤35°C, 35–37°C and >37°C |
| Ali et al., 2025 [36] | We quantified the cumulative relative risk over lags 0–14 days, comparing the 99th percentile of daily minimum temperature with the 50th percentile. (Data analysis) | 50^th^ percentile | Mortality risk: at the 99^th^ percentile. |
| Chu et al., 2025 [37] | We calculated the MMT and estimated cold- and heat-attributable mortality for temperatures below and above the MMT, respectively. (Statistical Analysis) | MMT (county-specific) | Mortality risk: at the 90^th^, 95^th^ and the 99^th^ percentiles. Attributable mortality: >MMT. |
| Frasch et al., 2025 [38] | Extreme cold and heat were defined at the 1st and 99th percentiles of daily mean temperature, respectively. (§2.1.2) The reference temperature was selected at the minimum relative risk between the 50th and 98th percentiles. If this value fell at the 99th percentile, the median served as the reference. (§2.2.2) Excess emergency admissions and fatal emergency admissions were estimated for the 1st and 99th temperature percentiles relative to the minimum-risk temperature for each outcome. (§2.2.3) | MMT (89th percentile) | Mortality risk: at the 99^th^ percentile. |
| Lu et al., 2025 [39] | Mortality odds ratios compared the region-specific 97.5th percentile of daily mean temperature, defining extreme high temperature, with the MMT. (Statistical analysis) | MMT (region-specific) | Mortality risk: at the 97.5^th^ percentile. |
| Sieck et al., 2025 [40] | Predictions were centred at the region-specific median temperature during the warm months. The main analysis estimated mortality risk at the region-specific 95th percentile. (Statistical analysis) Sensitivity analyses compared the 99th percentile with the median temperature. (Sensitivity analyses) | 50^th^ percentile (region-specific) | Mortality risk: at the 95^th^ percentile. |
| Zhang et al., 2025 [41] | The reference temperature was the daily mean temperature associated with the lowest estimated mortality risk, approximately 10 °C. Moderately high and extremely high daily mean temperatures were defined at the 90th percentile (28 °C) and the 97.5th percentile (32 °C), respectively. (Results) | MMT (10 °C) | Mortality risk: moderate heat at the 90^th^ percentile, extreme heat at the 97.5^th^ percentile. |

**References for studies included**

1. Lin YK, Chang CK, Li MH, Wu YC, Wang YC: High-temperature indices associated with mortality and outpatient visits: characterizing the association with elevated temperature. Sci Total Environ 2012, 427-428:41-49.

2. Chen R, Wang C, Meng X, Chen H, Thach TQ, Wong CM, Kan H: Both low and high temperature may increase the risk of stroke mortality. Neurology 2013, 81:1064-1070.

3. Ma W, Chen R, Kan H: Temperature-related mortality in 17 large Chinese cities: how heat and cold affect mortality in China. Environ Res 2014, 134:127-133.

4. Ng CF, Ueda K, Takeuchi A, Nitta H, Konishi S, Bagrowicz R, Watanabe C, Takami A: Sociogeographic variation in the effects of heat and cold on daily mortality in Japan. J Epidemiol 2014, 24:15-24.

5. Petkova EP, Gasparrini A, Kinney PL: Heat and mortality in New York City since the beginning of the 20th century. Epidemiology 2014, 25:554-560.

6. de' Donato FK, Leone M, Scortichini M, De Sario M, Katsouyanni K, Lanki T, Basagana X, Ballester F, Astrom C, Paldy A, et al.: Changes in the Effect of Heat on Mortality in the Last 20 Years in Nine European Cities. Results from the PHASE Project. Int J Environ Res Public Health 2015, 12:15567-15583.

7. Gasparrini A, Guo Y, Hashizume M, Lavigne E, Zanobetti A, Schwartz J, Tobias A, Tong S, Rocklov J, Forsberg B, et al.: Mortality risk attributable to high and low ambient temperature: a multicountry observational study. Lancet 2015, 386:369-375.

8. Lubczynska MJ, Christophi CA, Lelieveld J: Heat-related cardiovascular mortality risk in Cyprus: a case-crossover study using a distributed lag non-linear model. Environ Health 2015, 14:39.

9. Qiao Z, Guo Y, Yu W, Tong S: Assessment of Short- and Long-Term Mortality Displacement in Heat-Related Deaths in Brisbane, Australia, 1996-2004. Environ Health Perspect 2015, 123:766-772.

10. Cui Y, Yin F, Deng Y, Volinn E, Chen F, Ji K, Zeng J, Zhao X, Li X: Heat or Cold: Which One Exerts Greater Deleterious Effects on Health in a Basin Climate City? Impact of Ambient Temperature on Mortality in Chengdu, China. Int J Environ Res Public Health 2016, 13.

11. Achebak H, Devolder D, Ballester J: Heat-related mortality trends under recent climate warming in Spain: A 36-year observational study. PLoS Med 2018, 15:e1002617.

12. Aboubakri O, Khanjani N, Jahani Y, Bakhtiari B: Attributable risk of mortality associated with heat and heat waves: A time-series study in Kerman, Iran during 2005-2017. J Therm Biol 2019, 82:76-82.

13. Deng C, Ding Z, Li L, Wang Y, Guo P, Yang S, Liu J, Wang Y, Zhang Q: Burden of non-accidental mortality attributable to ambient temperatures: a time series study in a high plateau area of southwest China. BMJ Open 2019, 9:e024708.

14. Luan G, Yin P, Wang L, Zhou M: Associations between ambient high temperatures and suicide mortality: a multi-city time-series study in China. Environ Sci Pollut Res Int 2019, 26:20377-20385.

15. Alahmad B, Shakarchi AF, Khraishah H, Alseaidan M, Gasana J, Al-Hemoud A, Koutrakis P, Fox MA: Extreme temperatures and mortality in Kuwait: Who is vulnerable? Sci Total Environ 2020, 732:139289.

16. Ellena M, Ballester J, Mercogliano P, Ferracin E, Barbato G, Costa G, Ingole V: Social inequalities in heat-attributable mortality in the city of Turin, northwest of Italy: a time series analysis from 1982 to 2018. Environ Health 2020, 19:116.

17. Sera F, Hashizume M, Honda Y, Lavigne E, Schwartz J, Zanobetti A, Tobias A, Iniguez C, Vicedo-Cabrera AM, Blangiardo M, et al.: Air Conditioning and Heat-related Mortality: A Multi-country Longitudinal Study. Epidemiology 2020, 31:779-787.

18. Saucy A, Ragettli MS, Vienneau D, de Hoogh K, Tangermann L, Schaffer B, Wunderli JM, Probst-Hensch N, Roosli M: The role of extreme temperature in cause-specific acute cardiovascular mortality in Switzerland: A case-crossover study. Sci Total Environ 2021, 790:147958.

19. Casas L, Cox B, Nemery B, Deboosere P, Nawrot TS: High temperatures trigger suicide mortality in Brussels, Belgium: A case-crossover study (2002-2011). Environ Res 2022, 207:112159.

20. de Schrijver E, Bundo M, Ragettli MS, Sera F, Gasparrini A, Franco OH, Vicedo-Cabrera AM: Nationwide Analysis of the Heat- and Cold-Related Mortality Trends in Switzerland between 1969 and 2017: The Role of Population Aging. Environ Health Perspect 2022, 130:37001.

21. Ellena M, Ballester J, Costa G, Achebak H: Evolution of temperature-attributable mortality trends looking at social inequalities: An observational case study of urban maladaptation to cold and heat. Environ Res 2022, 214:114082.

22. Ingole V, Sheridan SC, Juvekar S, Achebak H, Moraga P: Mortality risk attributable to high and low ambient temperature in Pune city, India: A time series analysis from 2004 to 2012. Environ Res 2022, 204:112304.

23. Lee W, Choi M, Bell ML, Kang C, Jang J, Song I, Kim YO, Ebi K, Kim H: Effects of urbanization on vulnerability to heat-related mortality in urban and rural areas in South Korea: a nationwide district-level time-series study. Int J Epidemiol 2022, 51:111-121.

24. Roca-Barcelo A, Fecht D, Pirani M, Piel FB, Nardocci AC, Vineis P: Trends in Temperature-associated Mortality in Sao Paulo (Brazil) between 2000 and 2018: an Example of Disparities in Adaptation to Cold and Heat. J Urban Health 2022, 99:1012-1026.

25. Achebak H, Rey G, Lloyd SJ, Quijal-Zamorano M, Fernando Mendez-Turrubiates R, Ballester J: Drivers of the time-varying heat-cold-mortality association in Spain: A longitudinal observational study. Environ Int 2023, 182:108284.

26. Quilty S, Jupurrurla NF, Lal A, Matthews V, Gasparrini A, Hope P, Brearley M, Ebi KL: The relative value of sociocultural and infrastructural adaptations to heat in a very hot climate in northern Australia: a case time series of heat-associated mortality. Lancet Planet Health 2023, 7:e684-e693.

27. Ragettli MS, Saucy A, Fluckiger B, Vienneau D, de Hoogh K, Vicedo-Cabrera AM, Schindler C, Roosli M: Explorative Assessment of the Temperature-Mortality Association to Support Health-Based Heat-Warning Thresholds: A National Case-Crossover Study in Switzerland. Int J Environ Res Public Health 2023, 20.

28. Shrikhande SS, Pedder H, Roosli M, Dalvie MA, Lakshmanasamy R, Gasparrini A, Utzinger J, Cisse G: Non-optimal apparent temperature and cardiovascular mortality: the association in Puducherry, India between 2011 and 2020. BMC Public Health 2023, 23:291.

29. Tobias A, Madaniyazi L, Gasparrini A, Armstrong B: High Summer Temperatures and Heat Stroke Mortality in Spain. Epidemiology 2023, 34:892-896.

30. Alahmad B, Khraishah H, Kamineni M, Roye D, Papatheodorou SI, Vicedo-Cabrera AM, Guo Y, Lavigne E, Armstrong B, Sera F, et al.: Extreme Temperatures and Stroke Mortality: Evidence From a Multi-Country Analysis. Stroke 2024, 55:1847-1856.

31. Alwadi Y, Al-Delaimy WK, Abdulla F, Koutrakis P, Alahmad B: A 19-year analysis of hot and cold temperature burdens on mortality in Amman, Jordan. Sci Total Environ 2024, 951:175624.

32. Psistaki K, Kouis P, Michanikou A, Yiallouros PK, Papatheodorou SI, Paschalidou A: Temporal trends in temperature-related mortality and evidence for maladaptation to heat and cold in the Eastern Mediterranean region. Sci Total Environ 2024, 943:173899.

33. Ragettli MS, Fluckiger B, Vienneau D, Domingo-Irigoyen S, Koschenz M, Roosli M: Vulnerability to heat-related mortality and the effect of prevention measures: a time-stratified case-crossover study in Switzerland. Swiss Med Wkly 2024, 154:3410.

34. Wicki B, Fluckiger B, Vienneau D, de Hoogh K, Roosli M, Ragettli MS: Socio-environmental modifiers of heat-related mortality in eight Swiss cities: A case time series analysis. Environ Res 2024, 246:118116.

35. Yi W, Bach A, Tong S, Cheng J, Yang J, Zheng H, Ho HC, Song J, Pan R, Su H, et al.: Quantifying the historical and future heat-related mortality above the heat alert thresholds of the inaugural Chinese national heat-health action plan. Environ Res 2024, 262:119869.

36. Ali EA, Aerts R, Vaes B, Scheerens C, Beerten SG, Duarte E, Van Pottelbergh G: The overlapping impacts of heat and COVID-19 on mortality in Flanders, Belgium: a time-stratified case-crossover analysis. Sci Rep 2025, 15:44275.

37. Chu L, Dubrow R, Chen K: Heat- and Cold-Related Mortality Burden in the US From 2000 to 2020. JAMA Netw Open 2025, 8:e2542269.

38. Frasch JJ, Konig HH, Konnopka C: Effects of extreme temperature on morbidity, mortality, and case severity in German emergency care. Environ Res 2025, 270:121021.

39. Lu W, Sun H, Xu R, Shi C, Wei J, Liu Y, Zhou Y: Extreme Heat Exposure and COPD Mortality: Insights from Sleep Time. Environ Sci Technol 2025, 59:12074-12082.

40. Sieck NE, Liang M, Song H, He H, Raimann JG, Cruz R, Salawitch RJ, Sapkota AR, Maddux FW, Usvyat LA, et al.: Risk of hospitalization and mortality across US climate regions following extreme heat exposure in patients with end-stage kidney disease (ESKD) receiving in-center hemodialysis: a space-time-stratified case-crossover analysis. Environ Health 2025, 25:3.

41. Zhang T, Xie H, Zhang X, Ni J, Li G, Wu H, Zhu J, Pan F: Impact of non optimal ambient temperature and temperature variation on the mortality of cardiovascular diseases: a research in Wuhu, China. Int J Biometeorol 2025, 69:1701-1713.
